# Risk factors associated with indoor transmission during home quarantine of COVID-19 patients

**DOI:** 10.3389/fpubh.2023.1170085

**Published:** 2023-05-11

**Authors:** Yang Liu, Yan-Hua Chai, Yi-Fan Wu, Yu-Wei Zhang, Ling Wang, Ling Yang, Yi-Han Shi, Le-Le Wang, Li-Sha Zhang, Yan Chen, Rui Fan, Yu-Hua Wen, Heng Yang, Li Li, Yi-Han Liu, Hui-Zhen Zheng, Ji-Jin Jiang, Hao Qian, Ru-Jia Tao, Ye-Chang Qian, Ling-Wei Wang, Rong-Chang Chen, Jin-Fu Xu, Chen Wang

**Affiliations:** ^1^Institute of Respiratory Medicine, School of Medicine, Tongji University, Shanghai, China; ^2^Department of Respiratory Medicine, Baoshan District Hospital of Integrated Traditional Chinese and Western Medicine, Shanghai, China; ^3^Shenzhen Institute of Respiratory Disease, Shenzhen People’s Hospital (The Second Clinical Medical College, Jinan University; The First Affiliated Hospital, Southern University of Science and Technology), Shenzhen, China; ^4^Shenzhen Clinical Research Centre for Respirology, Shenzhen People’s Hospital, Shenzhen, China; ^5^National Center for Respiratory Medicine, Beijing, China; ^6^National Clinical Research Center for Respiratory Disease, Beijing, China; ^7^Institute of Respiratory Medicine, Chinese Academy of Medical Sciences, Peking Union Medical College, Beijing, China

**Keywords:** COVID-19, omicron, indoor transmission, risk factor, home quarantine

## Abstract

**Purpose:**

The study aimed to identify potential risk factors for family transmission and to provide precautionary guidelines for the general public during novel Coronavirus disease 2019 (COVID-19) waves.

**Methods:**

A retrospective cohort study with numerous COVID-19 patients recruited was conducted in Shanghai. Epidemiological data including transmission details, demographics, vaccination status, symptoms, comorbidities, antigen test, living environment, residential ventilation, disinfection and medical treatment of each participant were collected and risk factors for family transmission were determined.

**Results:**

A total of 2,334 COVID-19 patients participated. Compared with non-cohabitation infected patients, cohabitated ones were younger (*p* = 0.019), more commonly unvaccinated (*p* = 0.048) or exposed to infections (*p* < 0.001), and had higher rates of symptoms (*p* = 0.003) or shared living room (*p* < 0.001). Risk factors analysis showed that the 2019-nCov antigen positive (OR = 1.86, 95%CI 1.40–2.48, *p* < 0.001), symptoms development (OR = 1.86, 95%CI 1.34–2.58, *p* < 0.001), direct contact exposure (OR = 1.47, 95%CI 1.09–1.96, *p* = 0.010) were independent risk factors for the cohabitant transmission of COVID-19, and a separate room with a separate toilet could reduce the risk of family transmission (OR = 0.62, 95%CI 0.41–0.92, *p* = 0.018).

**Conclusion:**

Patients showing negative 2019-nCov antigen tests, being asymptomatic, living in a separate room with a separate toilet, or actively avoiding direct contact with cohabitants were at low risk of family transmission, and the study recommended that avoiding direct contact and residential disinfection could reduce the risk of all cohabitants within the same house being infected with COVID-19.

## 1. Introduction

Coronavirus disease 2019 (COVID-19) is a highly contagious viral disease caused by severe acute respiratory syndrome coronavirus 2 (SARS-CoV-2), which had a catastrophic impact on global health, resulting in 656 million confirmed cases and a death toll up to 6.67 million worldwide till January 04, 2023 (Data from 2019ncov.chinacdc.cn) (1–5). The variant strain of Omicron is less virulent, but evolves toward highly contagious (6–9). Shanghai, the biggest city of China with a population of 25 million, has been suffering a wave of sub-lineage of the Omicron variant. Due to its high transmission rate, the pandemic has caused a dramatic increase in the number of confirmed cases. According to the Shanghai Municipal Health Commission, as of May 4, 2022, 593 336 cases have been identified, and 503 people have died with or from COVID-19 (10). The first co-infection case with BA.5.2.48 and BF.7.14 has recently been reported in China (11).

Many COVID-19 cases only showed mild symptoms or were asymptomatic, but a previous study showed us the transmission potential of asymptomatic patients (12). Thus, to limit the spread of the disease, there is an urgent need for an evolutionary strategy to manage COVID-19 patients. Studies had revealed that isolation of infected persons, mask use at home, disinfection and keeping social distancing were critical to reducing SARS-CoV-2 transmission in household settings (13–15). However, these had not been received much attention in the public health practice and we believed it deserved to be highlighted again.

Considering that the home may be the smallest unit of population aggregation, scientific home quarantine is important for epidemic prevention to reduce the consumption of medical resources during the pandemic (16–19). The WHO-China Joint Mission on COVID-19 urged prioritization of studies on risk factors for household transmission (20). Therefore, taking advantage of the event of Shanghai in 2022, this study aimed to analyze the exposure sources and indoor transmission of 2,334 infected patients during home quarantine prior to shelter admission, to identify potential factors affecting home quarantine efficiency, and ultimately to provide precautionary guidelines for the general public during COVID-19 waves.

## 2. Materials and methods

### 2.1. Study design and participants

A retrospective cohort study was conducted from 10 March 2022 to 30 April 2022. Patients who were confirmed with COVID-19 by positive nucleic acid test (a positive nucleic acid was defined as Ct value<35 (21)) and were transmitted to Fangcang shelter hospital were enrolled in this study. Relevant information including demographic data, exposure sources and transmission of each participant during home quarantine period before going to the shelter hospital were collected via telephone interviews and questionnaires. And potential factors affecting home quarantine efficiency were explored. The study was approved by the Research Ethics Committee of Shanghai Pulmonary Hospital (IRB number: L22–236). Written informed consents were waived by the Ethics Commission of the designated hospitals because of retrospectively study related to this emerging public health.

### 2.2. Data collection

A standardized data collection spreadsheet was designed to obtain patients’ data from telephone interview and questionnaires (data in Supplementary File 2). All data collections were performed according to standardized protocol by researchers involved in this study. We defined cohabitation infection as the patient who became ill after direct contact with infected cohabitants, while non-cohabitation infection means that other cohabitants were not infected when the patient became infected, that is, she/he was the first person to be infected in the living environment. Two investigators independently reviewed the data collection forms to double check the validity of the data. Epidemiological data (upstream exposure source information, number of infected persons, downstream infected persons’ information, etc.), demographics (age, gender, BMI, occupation, educational level, vaccination information), symptoms onset (respiratory, gastrointestinal, neurological, etc.), comorbidities, 2019-nCov antigen test results, living environment, residential ventilation and disinfection, medical treatment were obtained for in-depth analysis.

### 2.3. Outcomes

The proportion of symptomatic and asymptomatic COVID-19 patients and differential indicators between the two groups were evaluated. The sensitivity of the 2019-nCov antigen test and differential indicators between patients with positive antigen test and negative antigen test were determined. Comparisons between non-cohabitation infection patients and cohabitated infection patients were performed and analyzed, and a household transmission rate of the epidemic was reported. Risk factors for household transmission of SARS-CoV-2 Omicron variant during home quarantine were clarified.

### 2.4. Statistical analysis

The Kolmogorov–Smirnov test was applied for analyzing the distribution of quantitative variables. Quantitative data were presented as median (interquartile range) for non-normally distributed variables or mean (standard deviation) for normally distributed variables and qualitative data were presented as frequencies (percentages). Independent group t-test was applied to analyze quantitative variables that were normally distributed and homoscedasticity, and the Mann–Whitney U test was applied to analyze quantitative variables that were non-normally distributed or not homoscedasticity. Qualitative variables between the two groups were compared by using Chi-square test or Fisher’s exact test. In the multivariate analysis, the logistic regression was used to determine risk factors associated with household transmission of SARS-CoV-2 Omicron variant. Variables with significance level *p* < 0.1 in the univariate analysis were preliminary screened out and were entered into the multivariate regression model. The odds ratio (OR) and 95% confidence interval (CI) were calculated for the independent variables. For all analyses, *p* < 0.05 was considered statistically significant. All statistical analyses and diagramming were performed by SPSS (version 23.0, IBM), GraphPad Prism (version 8.0, GraphPad Software Inc), Origin Pro (version 2016, OriginLab) softwares.

## 3. Results

### 3.1. Demographic characteristics and symptom distribution

A total of 2,334 confirmed COVID-19 patients were included. Patients were aged 1–91 years with a median age of 43 years (31–54), and 1,286 (55.1%) were male (Table 1). On the basis of their presentations prior to the examined day, 616 (26.4%) patients without any clinical signs or symptoms were classified as asymptomatic, while 1718 (73.6%) were classified as symptomatic. Compared with asymptomatic patients, symptomatic cases had higher rates of 2019-nCov antigen positive (283 [52.2%] vs. 1,249 [76.2%], *p* < 0.001) and household transmission (63 [9.9%] vs. 344 [20.0%], *p* < 0.001). A total of 2,181 (93.4%) patients completed the 2019-nCov antigen test at the same time as the nucleic acid test and the sensitivity of the 2019-nCov antigen test was 70.2% (1532) in our study. Compared with 2019-nCov antigen-negative patients, the proportion of 2019-nCov antigen-positive patients who had symptoms (390 [60.1%] vs. 1,249 [81.5%], *p* < 0.001), medical treatment (352 [54.2%] vs. 1,108 [72.3%], p < 0.001), and downstream cohabitants transmission (71 [10.9%] vs. 315 [20.6%], p < 0.001) were significantly higher (Supplementary Table 1).

**Table 1:** Differential characteristics between asymptomatic and symptomatic COVID-19 cases.

Variables	Number of valid data	Total	Symptomatic	Asymptomatic	*p*
			1718	616	
Age	2,334				0.1
~30		554 (23.7%)	421 (24.5%)	133 (21.6%)	
31–59		1,411 (60.5%)	1,040 (60.5%)	371 (60.2%)	
60~		369 (15.8%)	257 (15.0%)	112 (18.2%)	
Sex (Male)	2,334	1,286 (55.1%)	884 (51.5%)	402 (65.3%)	<0.001
BMI	2,270	23.45 (21.07–25.95)	23.44 (21.09–25.89)	23.53 (20.96–26.17)	0.59
Educational level	2,275				<0.001
Junior high and below		1,261 (55.4%)	906 (53.5%)	355 (61.0%)	
High school		594 (26.1%)	441 (26.0%)	153 (26.3%)	
Undergraduate		373 (16.4%)	308 (18.2%)	65 (11.2%)	
Graduate		47 (2.1%)	38 (2.2%)	9 (1.5%)	
Number of vaccinations	2,311				0.526
0		276 (11.9%)	202 (11.8%)	74 (12.4%)	
1		81 (3.5%)	58 (3.4%)	23 (3.9%)	
2		802 (34.7%)	610 (35.5%)	192 (32.3%)	
3		1,152 (49.8%)	846 (49.3%)	306 (51.4%)	
Comorbidity	2,299				0.011
None		1866 (81.2%)	1,345 (79.9%)	521 (84.6%)	
≥1		433 (18.8%)	338 (20.1%)	95 (15.4%)	
2019-nCov antigen positive	2,181	1,532 (70.2%)	1,249 (76.2%)	283 (52.2%)	<0.001
Medical treatment	2,334				<0.001
None		798 (34.2%)	405 (23.6%)	393 (63.8%)	
≥1		1,536 (65.8%)	1,313 (76.4%)	223 (36.2%)	
Cohabitants transmission	2,334				<0.001
None		1929 (82.6%)	1,374 (80.0%)	555 (90.1%)	
1		225 (9.6%)	188 (10.9%)	37 (6.0%)	
≥ 2		180 (7.7%)	156 (9.1%)	24 (3.9%)	

Quantitative data were summarized as median (IQR) for non-normally distribute variables and qualitative data were presented as *n* (percentage).

### 3.2. Differential characteristics of COVID-19 patients with cohabitated infection and non-cohabitation infection

**Figure 1:**
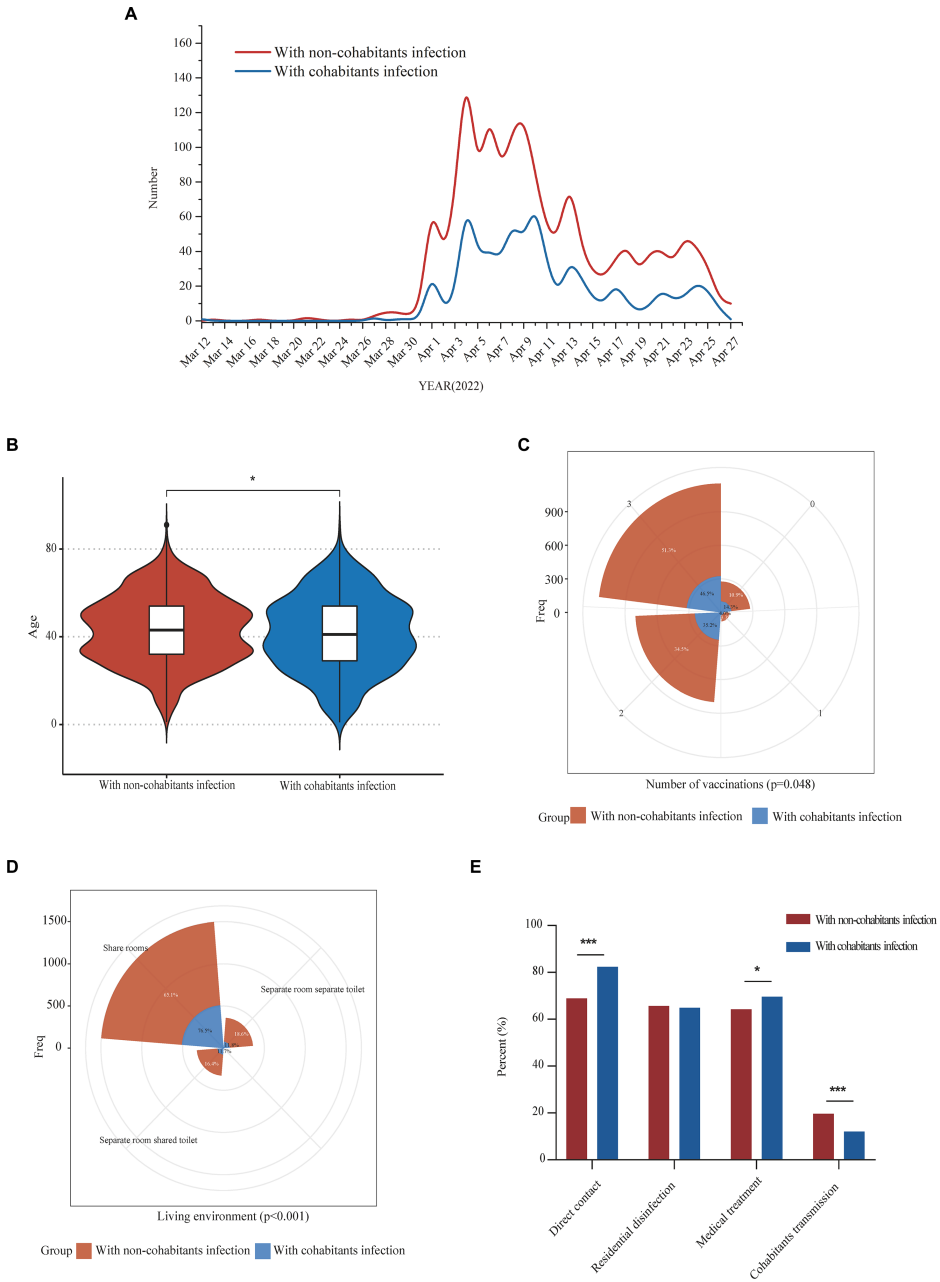
Epidemiological characteristics comparison of COVID-19 patients with infectious exposure from cohabitants and non-cohabitants. Panel **(A)** showed the daily numbers of COVID-19 patients with infectious exposure from cohabitants and non-cohabitants from 12 March 2022 to 27 April 2022; Comparisons of differences in age, vaccination frequency and living environment between the two groups were shown in panel **(B–D)** respectively; Differences in the proportion of direct contact exposure, residential disinfection, medical treatment and downstream cohabitants transmission between the two groups were shown in panel **(E)**. *p < 0.05; **p < 0.01; ***p < 0.001.

In this study, we investigated the upstream potential exposure factors of patients and found that of the 2,334 patients enrolled, 698 (29.9%) patients were infected from their cohabitants, and 1,636 (70.1%) patients were infected from non-cohabitants. The diagnostic time line for the two groups was shown in Figure 1A. Compared with patients with non-cohabitation infection, patients with cohabitated infection were younger (43 [32–54] vs. 41 [29–54], *p* = 0.019), more common being unvaccinated (176 [10.9%] vs. 100 [14.3%], *p* = 0.048) and having direct contact exposure (1,054 [68.9%] vs. 552 [82.4%], *p* < 0.001), had a higher rate of symptoms (1,175 [71.8%] vs. 543 [77.8%], *p* = 0.003) and needed more medical treatment (1,050 [64.2%] vs. 486 [69.6%], *p* = 0.011), as well as having a higher proportion of sharing living room (989 [65.1%] vs. 511 [76.5%], *p* < 0.001) (Table 2, Figures 1B–E). We further analyzed the age of the upstream transmission source population, the enrolled patient population, and the downstream infected population, and found that downstream infected patients were significantly younger than upstream population and all enrolled patients (38 [25–54] vs. 46 [33–55], *p* < 0.001; 38 [25–54] vs. 43 [31–54], *p* < 0.001) (Supplementary Figure S1). In addition, the household transmission rate of patients with cohabitated infection was lower than that of patients with non-cohabitation infection (84 [12.0%] vs. 321 [19.6%], *p* < 0.001) (Table 2).

**Table 2:** Comparative analysis of baseline characteristics of COVID-19 patients with infectious exposure from cohabitants and non-cohabitants.

Variables	Number of valid data	Total	With non-cohabitants infection	With cohabitants infection	*p*
			1,636	698	
Age	2,334				0.003
~30		554 (23.7%)	357 (21.8%)	197 (28.2%)	
31–59		1,411 (60.5%)	1,020 (62.3%)	391 (56.0%)	
60~		369 (15.8%)	259 (15.8%)	110 (15.8%)	
Sex (Male)	2,334	1,286 (55.1%)	894 (54.6%)	392 (56.2%)	0.5
BMI	2,270	23.45 (21.07–25.95)	23.53 (21.09–25.95)	23.44 (20.96–25.99)	0.585
Educational level	2,275				0.763
Junior high and below		1,261 (55.4%)	879 (55.4%)	382 (55.5%)	
High school		594 (26.1%)	421 (26.5%)	173 (25.1%)	
Undergraduate		373 (16.4%)	253 (15.9%)	120 (17.4%)	
Graduate		47 (2.1%)	34 (2.1%)	13 (1.9%)	
Symptoms	2,334				0.003
None		616 (26.4%)	461 (28.2%)	155 (22.2%)	
≥1		1718 (73.6%)	1,175 (71.8%)	543 (77.8%)	
Number of vaccinations	2,311				0.048
0		276 (11.9%)	176 (10.9%)	100 (14.3%)	
1		81 (3.5%)	53 (3.3%)	28 (4.0%)	
2		802 (34.7%)	557 (34.5%)	245 (35.2%)	
3		1,152 (49.8%)	828 (51.3%)	324 (46.5%)	
Comorbidity	2,299				0.943
None		1866 (81.2%)	1,309 (81.2%)	557 (81.8%)	
≥1		433 (18.8%)	303 (18.8%)	130 (18.9%)	
2019-nCov antigen positive	2,181	1,532 (70.2%)	1,082 (71.1%)	450 (68.3%)	0.188
Living environment	2,188				<0.001
Separate room separate toilet		361 (16.5%)	282 (18.6%)	79 (11.8%)	
Separate room shared toilet		327 (14.9%)	249 (16.4%)	78 (11.7%)	
Share rooms		1,500 (68.6%)	989 (65.1%)	511 (76.5%)	
Residential ventilation (Yes)	2,293	2025 (88.3%)	1,403 (87.7%)	622 (89.8%)	0.157
Direct contact (Yes)	2,200	1,606 (73.0%)	1,054 (68.9%)	552 (82.4%)	<0.001
Residential disinfection (Yes)	2,266	1,481 (65.4%)	1,034 (65.6%)	447 (64.9%)	0.751
Medical treatment	2,334				0.011
None		798 (34.2%)	586 (35.8%)	212 (30.4%)	
≥1		1,536 (65.8%)	1,050 (64.2%)	486 (69.6%)	
Cohabitants transmission	2,334				<0.001
None		1929 (82.6%)	1,315 (80.4%)	614 (88.0%)	
1		225 (9.6%)	184 (11.2%)	41 (5.9%)	
≥2		180 (7.7%)	137 (8.4%)	43 (6.2%)	

Quantitative data were summarized as median (IQR) for non-normally distribute variables and qualitative data were presented as n (percentage); *p < 0.05 compared with control group.

### 3.3. Independent factors associated with household transmission of SARS-CoV-2 omicron variant during home quarantine

**Figure 2:**
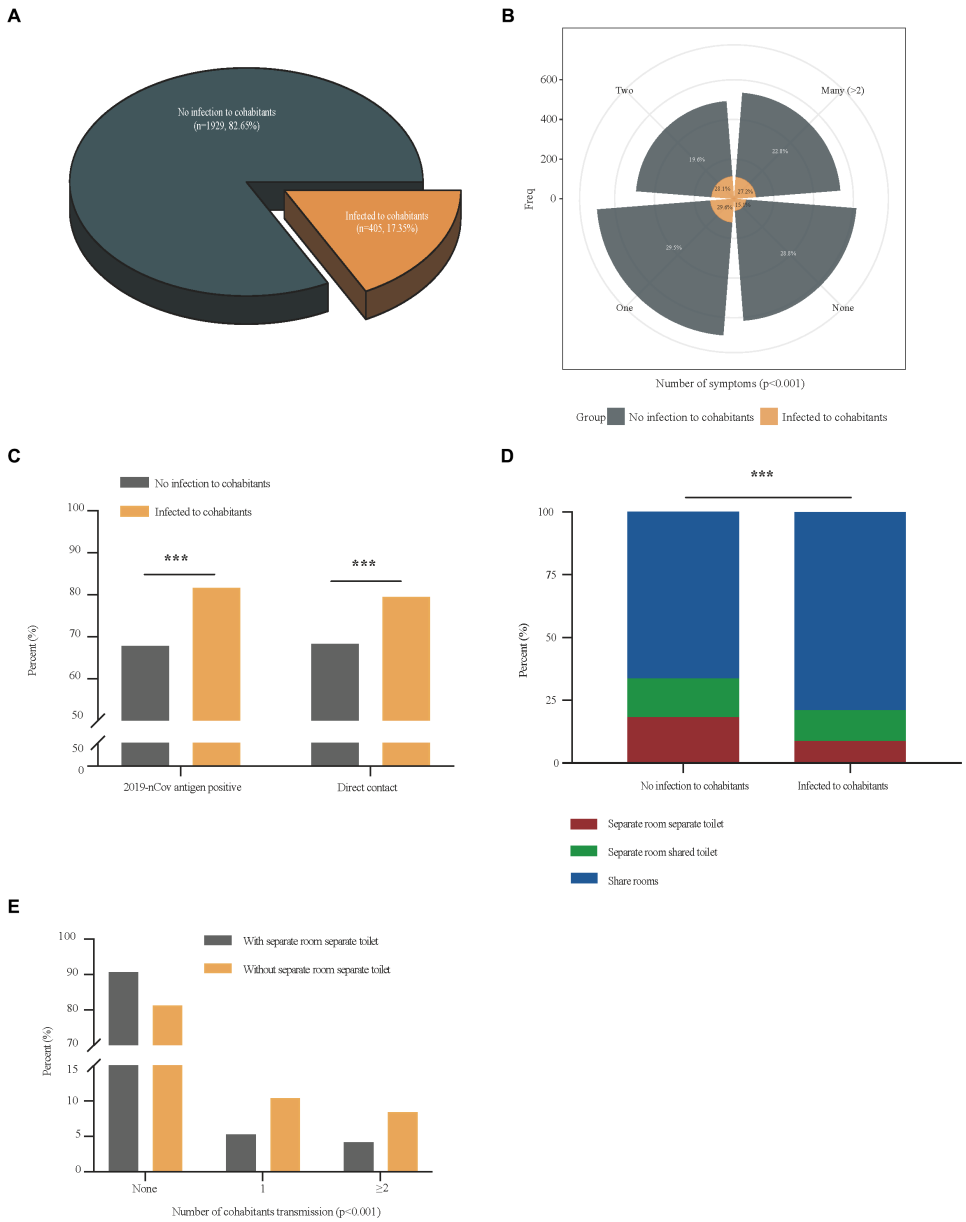
Comparisons of differential factors of COVID-19 patients with and without cohabitation transmission. Panel **(A)** showed the proportion of patients who transmitted the disease to their cohabitants in total patients; Differences in the number of symptoms, and the proportion of 2019-nCov antigen positive, direct contact exposure and living environments between COVID-19 patients with cohabitants transmission and those without cohabitants transmission were shown in panel **(B–D)**; Differences in the number of cohabitants transmission between COVID-19 patients with separate room separate toilet and those without separate room separate toilet was shown in panel **(E)**. ***p < 0.001.

There were 405 (17.4%) cases in which patients transmitted the disease to their cohabitants (Figure 2A). The multivariate analysis of independent factors associated with household transmission of SARS-CoV-2 Omicron variant during home quarantine was showed in Table 3. Independent risk factors including 2019-nCov antigen positive (OR = 1.86, 95%CI 1.40–2.48, *p* < 0.001), symptoms development (OR = 1.86, 95%CI 1.34–2.58, *p* < 0.001), direct contact exposure (OR = 1.47, 95%CI 1.09–1.96, *p* = 0.010) were associated with increased household transmission of the disease, while having a separate room with a separate toilet could reduce the risk of household transmission during home quarantine (OR = 0.62, 95%CI 0.41–0.92, *p* = 0.018). Compared with patients who had no infection to cohabitants, patients that transmitted the disease to their cohabitants had higher rates of symptoms (1,374 [71.2%] vs. 344 [84.9%], *p* < 0.001) (Figure 2B), 2019-nCov antigen positive (1,217 [67.8%] vs. 315 [81.6%], *p* < 0.001), direct contact exposure (1,285 [68.3%] vs. 321 [79.5%], *p* < 0.001) (Figure 2C), as well as a higher proportion of patients with living environment of a sharing room (1,193 [66.3%] vs. 307 [78.9%], *p* < 0.001) (Figure 2D). Considering that living environment significantly affected the spread of the disease among the cohabitants during home quarantine, we divided all patients into two groups with or without a separate room and a separate toilet, and analyzed the difference in the household transmission rates between the two groups. Results showed that the downstream household transmission rate of patients without a separate room and a separate toilet was significantly increased compared with that of patients with a separate room and a separate toilet (371 [18.8%] vs. 34 [9.4%], *p* < 0.001) (Figure 2E).

**Table 3:** Multivariate analysis of independent factors associated with cohabitants transmission of COVID-19.

Factors	Unadjusted OR (95%CI)	*p*	Adjusted OR (95%CI)	*p*
Age	1.008 (1.001–1.015)	0.018	1.007 (0.999–1.015)	0.078
BMI	1.009 (0.981–1.037)	0.533		
Educational level	0.982 (0.860–1.120)	0.784		
Number of vaccinations	1.036 (0.927–1.158)	0.529		
Comorbidity (Yes)	1.356 (1.044–1.762)	0.022	1.181 (0.870–1.602)	0.286
2019-nCov antigen positive	2.107 (1.599–2.776)	<0.001	1.861 (1.397–2.480)	<0.001
Symptoms (Yes)	2.278 (1.705–3.043)	<0.001	1.859 (1.338–2.583)	<0.001
Separate room separate toilet (Yes)	0.449 (0.310–0.651)	<0.001	0.616 (0.412–0.921)	0.018
Residential ventilation (Yes)	1.068 (0.760–1.500)	0.705		
Direct contact (Yes)	1.782 (1.373–2.311)	<0.001	1.465 (1.094–1.960)	0.010
Medical treatment (Yes)	1.587 (1.243–2.027)	<0.001	1.080 (0.822–1.419)	0.581
Residential disinfection (Yes)	0.812 (0.650–1.015)	0.068	0.925 (0.729–1.172)	0.516

Among 2,334 enrolled patients, there were 405 cases in which patients transmitted the disease to their cohabitants, and 1929 patients did not have cohabitants transmission. OR: Odd ratio; CI: Confidence interval.

### 3.4. Self-protection awareness of patients was closely related To whether all cohabitants within the same house were infected with SARS-CoV-2 omicron variant during home quarantine

Of the 2,334 patients enrolled in the study, all cohabitants of 811 (34.7%) patients were confirmed with COVID-19 during home quarantine. Important characteristics of these 811 patients were summarized in Supplementary Table S2. Patients were aged 1–89 years, with a median age of 41 years (30–54), and 479 (59.1%) were male. Among them, 386 (47.6%) patients had completed three doses of vaccination, 605 (74.6%) patients had symptoms, 138 (17.2%) patients had comorbidity, 532 (65.9%) patients had taken medical treatment, only 94 (12.5%) patients had a living environment of a separate room and a separate toilet, and up to 631 (78.3%) patients had direct contact exposure with their cohabitants. In this population, the positive rate of 2019-nCov antigen test of patients was 71.6% (544), and the cohabitants transmission rate of these patients was 21.6% (175). The multivariate results of independent factors associated with COVID-19 infection in all cohabitants within the same house during home quarantine were shown in Table 4. Direct contact exposure with cohabitants (OR = 1.36, 95%CI 1.09–1.71, *p* = 0.008) was an independent risk factor for the virus infection in all cohabitants within the same house during home quarantine, while residential disinfection could reduce this risk (OR = 0.78, 95%CI 0.63–0.95, *p* = 0.016).

**Table 4:** Multivariate analysis of independent factors associated with COVID-19 infection in all cohabitants within the same house during home quarantine.

Factors	Unadjusted OR (95%CI)	*p*	Adjusted OR (95%CI)	*p*
Age	0.998 (0.992–1.004)	0.493		
BMI	1.004 (0.979–1.029)	0.755		
Educational level	1.038 (0.924–1.165)	0.530		
Number of vaccinations	0.909 (0.823–1.004)	0.060	0.929 (0.840–1.027)	0.151
Comorbidity (Yes)	0.971 (0.754–1.249)	0.817		
2019-nCov antigen positive	1.166 (0.941–1.446)	0.160		
Symptoms (Yes)	1.206 (0.973–1.494)	0.088	1.163 (0.934–1.447)	0.177
Separate room separate toilet (Yes)	0.992 (0.737–1.336)	0.957		
Residential ventilation (Yes)	0.893 (0.664–1.201)	0.454		
Direct contact (Yes)	1.400 (1.120–1.750)	0.003	1.361 (1.086–1.707)	0.008
Medical treatment (Yes)	0.981 (0.803–1.200)	0.853		
Residential disinfection (Yes)	0.753 (0.615–0.923)	0.006	0.777 (0.632–0.954)	0.016

Among 2,334 enrolled patients, 1,684 cases were cohabiting, and all residents of 811 (48.2%) patients were confirmed with COVID-19. OR: Odd ratio; CI: Confidence interval.

## 4. Discussion

It may lead to secondary or multiple transmission of the COVID-19 if effective measures were not taken when families were infected. Studies showed that the outbreak of infection in a large population over a short time could easily lead to the emergence of new mutant strains. Minimizing the prevalence of SARS-CoV-2 will reduce chances of forming recombinant lineages with genetic combinations that may increase the adaptability of the virus potentially (6, 22). It is particularly important to protect vulnerable groups such as the older adults, children and pregnant women by reducing household transmission of the disease effectively. And the Lancet was calling for the creation of a strong resilient health system and international preparedness strategies to control the pandemic (23). Thus, our study analyzed and determined risk factors associated with household transmission of SARS-CoV-2 Omicron variant through a large-scale epidemiological survey of 2,234 patients. Based on the results, we found a group of patients who were at low risk of transmission. They were patients with 2019-nCov antigen negative, no relevant clinical symptoms, living in a separate room with a separate toilet, and actively avoiding direct contact with cohabitants.

SARS-CoV-2 is more transmissible in households than SARS-CoV and Middle East respiratory syndrome coronavirus (24). The long incubation period and high presymptomatic contagiousness of COVID-19 make transmission among family members a special risk (25, 26). Evidence on the spread of SARS-CoV-2 Omicron variant during home quarantine has been reported in different countries, while characteristics of family transmission of the COVID-19 pandemic and important factors associated with it were still poorly understood (5, 13, 27, 28). In this study, we observed that the household transmission rate of the Omicron variant was 17.4%, which was much lower than that reported in the United States (52.7%) and South Korea (50.0%) (13, 29). In addition, Madewell et al. systematically analyzed the household secondary attack rates of Omicron variant from seven foreign studies and found that the overall household secondary attack rate was 42.7% (35.4–50.4%) (30), which was also higher than that in Shanghai in 2022. The heterogeneity in household transmission rates in different regions may be due to differences in control measures, surveillance practices, and household crowding.

Studies had revealed that many factors could influence the secondary transmission of SARS-CoV-2 in households, including isolation of infected persons, mask use at home, disinfection and social distancing (13, 14). In addition to these factors, our study showed that 2019-nCov antigen positive and symptoms development were independent risk factors for family transmission of the disease. Moreover, the study recommended that avoiding direct contact and residential disinfection could reduce the risk of all cohabitants within the same house being infected with SARS-CoV-2 Omicron variant. These will inform precautionary guidelines for families to reduce indoor transmission in areas where there is high community transmission for COVID-19.

It had been reported that viral load in asymptomatic patients was comparable to that in symptomatic patients, suggesting the transmission potential of asymptomatic patients (12, 31). However, Li et al. found that asymptomatic patients were less likely to infect others than symptomatic cases, and symptomatic cases were more infectious during the incubation period than those during the symptomatic period (20). These were consistent with our finding that symptomatic cases had higher rates of household transmission than that of asymptomatic patients. Antigen-based rapid tests have important diagnostic value early in the course of disease, which showed relatively high sensitivity in SARS-CoV-2 diagnosis in the early phase of infection (32). It had been reported that the average sensitivity of commercial antigen assays was higher in symptomatic compared to asymptomatic participants (33), and antigen-based rapid diagnostic test sensitivity was higher in the first 7 days after symptom onset than that in asymptomatic patients (34). Similarly, symptomatic patients had a higher proportion of positive 2019-nCov antigen testing than asymptomatic patients in this study. These may be related to the different courses of the disease in the two groups of patients when the antigen testing was performed. The above provided possible explanations why 2019-nCov antigen positive, symptomatic patients were at a higher risk of household transmission during home quarantine.

The spread of the novel coronavirus was associated with exposure to fomites, aerosols, and droplets, especially in the crowded or confined spaces (8, 35). It had been reported that the risk and probability of being caught by the indoor COVID-19 disease increased in time, particularly in the downstream of a localized infectious person (36). A previous study provided the evidence of the effectiveness of social distancing in preventing COVID-19 (14), which was consistent with our findings that direct contact exposure could increase the risk of home transmission, and the living conditions of a separate room with a separate toilet could protect the cohabitants from infection. Randomized clinical trials had shown that hand hygiene alone did not prevent respiratory transmissible viruses, but the combination of masks did work (37). Importantly, our study showed that, except for the uncontrollable conditions of the patient, it could greatly reduce the spread of the disease during home quarantine by improving hygiene measures such as avoiding direct contact with cohabitants and actively disinfecting the house, and providing separate living space for infected cohabitants, thereby greatly reducing the risk that all of the family members becoming infected.

In addition, we found that patients with cohabitation infection were younger than those with non-cohabitation infection. The phenomenon may be partially due to a lack of self-protect awareness, but the main reason should be that most older patients were responsible for the normal life of their families and were more likely to go out to the gathering places such as supermarkets or farmers’ markets to buy daily necessities, which increases the risk of infection. While most young patients were less active, and their infection mainly came from contacting with their infected cohabitants. Moreover, the living environment of these people was mainly of sharing room, and the proportion of isolated conditions with a separate room and a separate toilet was very small, making it difficult to avoid contact with infected cohabitants. These indicated that home quarantine conditions played a very important role in the spread of the epidemic within the household. It is noteworthy that the number of vaccinations was not an independent risk factor of home transmission in our study. Similarly, previous studies observed in some countries that, the vaccination offered less protect against the spread of disease, since some portions of vaccinated people were not totally immunized (38, 39).

Our study has several limitations. Telephone interviews have inherent limitations, including recall bias. In addition, we could not collect baseline information on uninfected cohabitants of enrolled patients, and therefore could not do a comparative analysis. Besides, the study could only define that the non-cohabitation infection originated from social contact, and could not exclude other infection risks.

In conclusion, important factors revealed in this study, including negative 2019-nCov antigen tests, absence of associated clinical symptoms, living in a separate room with a separate toilet, and active avoidance of direct contact with cohabitants, will inform precautionary guidelines for all families to reduce household transmission during the waves of COVID-19 pandemic, and the study recommended that avoiding direct contact and residential disinfection could reduce the risk of all cohabitants within the same house being infected with COVID-19.

## Data availability statement

The original contributions presented in the study are included in the article/Supplementary material, further inquiries can be directed to the corresponding authors.

## Ethics statement

The studies involving human participants were reviewed and approved by the Research Ethics Committee of Shanghai Pulmonary Hospital. Written informed consent for participation was not provided by the participants’ legal guardians/next of kin because: written informed consents were waived by the Ethics Commission of the designated hospitals because of retrospectively study related to this emerging public health.

## Author contributions

YL, Y-HC, and Y-FW: data analysis and these authors have contributed equally to this work. CW and J-FX: supervision, concept, and design. YL, Y-HC, Y-FW, Y-WZ, LW, LY, Y-HS, L-LW, L-SZ, YC, RF, Y-HW, HY, LL, Y-HL, H-ZZ, J-JJ, HQ, R-JT, and Y-CQ: acquisition and interpretation of data. YL, J-FX, and CW: drafting of the manuscript. L-WW, R-CC, J-FX, and CW: critical revision of the manuscript for important intellectual content and administrative, technical, or material support. All authors contributed to the article and approved the submitted version.

## Conflict of interest

The authors declare that the research was conducted in the absence of any commercial or financial relationships that could be construed as a potential conflict of interest.

## Publisher’s note

All claims expressed in this article are solely those of the authors and do not necessarily represent those of their affiliated organizations, or those of the publisher, the editors and the reviewers. Any product that may be evaluated in this article, or claim that may be made by its manufacturer, is not guaranteed or endorsed by the publisher.
