# Supplementary material for: Risk factors associated with indoor transmission during home quarantine of COVID-19 patients
Source: Front Public Health. 2023 May 11;11:1170085. doi: 10.3389/fpubh.2023.1170085 (PMC10213781; doi:10.3389/fpubh.2023.1170085)
Supplement: Supplementary file 1 [file Data_Sheet_1.PDF]

Supplementary table 1. Differential characteristics comparisons between 2019-nCov antigen-positive and negative COVID-19 patients

Variables	Number of valid data	Total	2019-nCov antigen positive 1532	2019-nCov antigen negative 649	*p*
Age	2181	42 (31-54)	42 (31-54)	43 (30-54)	0·705
Sex (Male)	2181	1211 (55·5%)	859 (56·1%)	352 (54·2%)	0·431
BMI	2136	23·48 (21·10-25·95)	23·51 (21·11-25·95)	23·44 (21·09-25·95)	0·513
Symptoms	2181				<0·001
None		542 (24·9%)	283 (18·5%)	259 (39·9%)	
≥1		1639 (75·1%)	1249 (81·5%)	390 (60·1%)	
Medical treatment	2181				<0·001
None		721 (33·1%)	424 (27·7%)	297 (45·8%)	
≥1		1460 (66·9%)	1108 (72·3%)	352 (54·2%)	
Cohabitants transmission	2181				<0·001
None		1795 (82·3%)	1217 (79·4%)	578 (89·1%)	
1		216 (9·9%)	175 (11·4%)	41 (6·3%)	
≥ 2		170 (7·8%)	140 (9·1%)	30 (4·6%)	

Note: Quantitative data were summarized as median (IQR) for non-normally distribute variables and qualitative data were presented as n (percentage); *p<0·05 compared with control group.

Supplementary table 2. Important characteristics of special cases in which all cohabitants were confirmed with COVID-19

Variables	Number of valid data	Total
Age	811	41 (30-54)
Sex (Male)	811	479 (59·1%)
BMI	809	23·44 (20·96-25·95)
Symptoms (Yes)	811	605 (74·6%)
Number of vaccinations	811	
0		104 (12·8%)
1		27 (3·3%)
2		294 (36·3%)
3		386 (47·6%)
Comorbidity (Yes)	801	138 (17·2%)
2019-nCov antigen positive	760	544 (71·6%)
Living environment	753	
Separate room separate toilet		94 (12·5%)
Separate room shared toilet		80 (10·6%)
Share rooms		579 (76·9%)
Residential ventilation (Yes)	808	708 (87·6%)
Direct contact (Yes)	806	631 (78·3%)
Residential disinfection (Yes)	806	507 (62·9%)
Medical treatment (Yes)	807	532 (65·9%)
Cohabitants transmission	811	
None		636 (78·4%)
1		91 (11·2%)
≥ 2		84 (10·4%)

Note: Quantitative data were summarized as median (IQR) for non-normally distribute variables and qualitative data were presented as n (percentage).

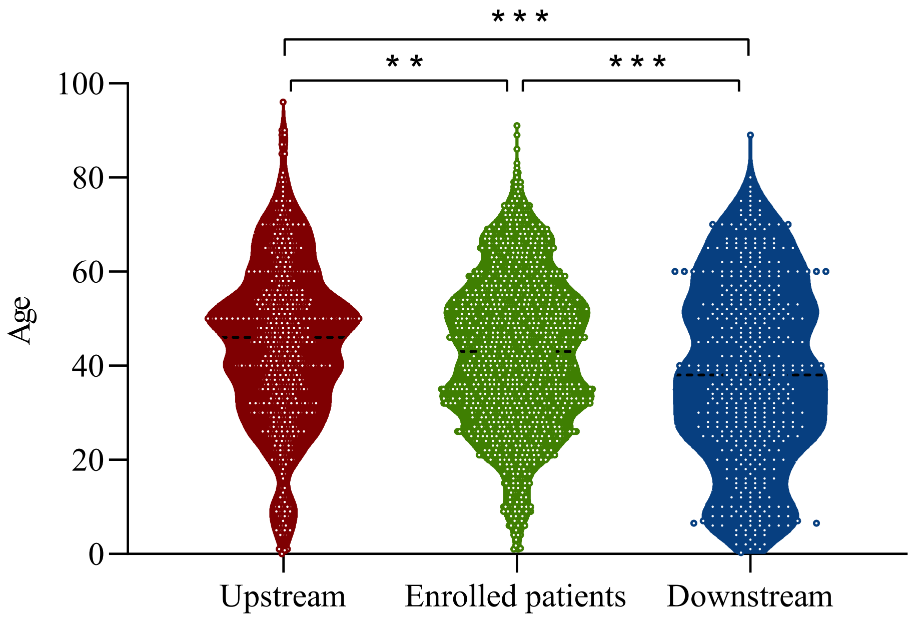

Supplementary fig 1. Age comparison of all enrolled COVID-19 patients and their upstream and downstream patients

Shown was the difference of age among all enrolled COVID-19 patients and their upstream and downstream patients. **: p<0·01, ***: p<0·001.
